# A microarray approach to identify genes involved in seed-pericarp cross-talk and development in peach

**DOI:** 10.1186/1471-2229-11-107

**Published:** 2011-06-16

**Authors:** Claudio Bonghi, Livio Trainotti, Alessandro Botton, Alice Tadiello, Angela Rasori, Fiorenza Ziliotto, Valerio Zaffalon, Giorgio Casadoro, Angelo Ramina

**Affiliations:** 1 Department of Environmental Agronomy and Crop Science, University of Padova, Legnaro (PD), Italy; 2 Department of Biology, University of Padova, Viale G. Colombo, 3 35121 Padova (PD), Italy

## Abstract

**Background:**

Field observations and a few physiological studies have demonstrated that peach embryogenesis and fruit development are tightly coupled. In fact, attempts to stimulate parthenocarpic fruit development by means of external tools have failed. Moreover, physiological disturbances during early embryo development lead to seed abortion and fruitlet abscission. Later in embryo development, the interactions between seed and fruit development become less strict. As there is limited genetic and molecular information about seed-pericarp cross-talk and development in peach, a massive gene approach based on the use of the μPEACH 1.0 array platform and quantitative real time RT-PCR (qRT-PCR) was used to study this process.

**Results:**

A comparative analysis of the transcription profiles conducted in seed and mesocarp (cv Fantasia) throughout different developmental stages (S1, S2, S3 and S4) evidenced that 455 genes are differentially expressed in seed and fruit. Among differentially expressed genes some were validated as markers in two subsequent years and in three different genotypes. Seed markers were a LTP1 (lipid transfer protein), a PR (pathogenesis-related) protein, a prunin and LEA (Late Embryogenesis Abundant) protein, for S1, S2, S3 and S4, respectively. Mesocarp markers were a RD22-like protein, a serin-carboxypeptidase, a senescence related protein and an Aux/IAA, for S1, S2, S3 and S4, respectively.

The microarray data, analyzed by using the HORMONOMETER platform, allowed the identification of hormone-responsive genes, some of them putatively involved in seed-pericarp crosstalk. Results indicated that auxin, cytokinins, and gibberellins are good candidates, acting either directly (auxin) or indirectly as signals during early development, when the cross-talk is more active and vital for fruit set, whereas abscisic acid and ethylene may be involved later on.

**Conclusions:**

In this research, genes were identified marking different phases of seed and mesocarp development. The selected genes behaved as good seed markers, while for mesocarp their reliability appeared to be dependent upon developmental and ripening traits. Regarding the cross-talk between seed and pericarp, possible candidate signals were identified among hormones.

Further investigations relying upon the availability of whole genome platforms will allow the enrichment of a marker genes repertoire and the elucidation of players other than hormones that are involved in seed-pericarp cross-talk (i.e. hormone peptides and microRNAs).

## Background

Peach fruit development is tightly connected to embryogenesis. Fruit growth displays a double sigmoid pattern in which four stages named S1, S2, S3 and S4 can be distinguished [1]. The early part of S1 is characterized by cell division and enlargement lasting about two weeks, followed by cell enlargement. The slowdown in growth that occurs at S1/S2 transition is followed by endocarp lignification (pit hardening), which lasts for 12-15 days from the middle of S2 to its end. S3 starts with a resumption of growth mainly due to cell enlargement, thus generating the second exponential phase. Maturation is completed by the end of S3 and followed by ripening (S4). The four fruit developmental phases are determined using a mathematical model based on first derivative of the growth curve [1]. Identification of the growth phases is important both for developmental studies and for precision farming. However, the only easily detectable event is the end of pit hardening marking the S2/S3 transition, because the phase length is affected by both genotype (early, middle and late ripening varieties) and environmental cues. A continuous growth model reassessment is therefore required. Accordingly, the identification of developmental phase organ-specific molecular markers would be of great importance for scientific and practical purposes.

Seed development, necessary for fruit set [2], is characterized by a fast endosperm growth that starts immediately after fertilization concurrently with the nucellus re-absorption, and lasts until the beginning of endocarp lignification, when the seed reaches its final size. At the end of pit hardening, seed volume is mainly made up of endosperm and the embryo is at the heart stage. Thereafter, embryo growth resumes and cotyledon development is paralleled by endosperm re-absorption. Seed maturation is characterized by lipids accumulations [3], synthesis of specific late embryogenesis abundant (LEA) proteins and dehydration. Attempts to stimulate parthenocarpic fruit development by hormone applications resulted as being ineffective. Moreover, seed abnormalities at the early stages of development (S1 and S1/S2 transition stages) lead to abortion and fruitlet abscission [4]. Later, (late S2, S3 and S4), the relationships between fruit development and embryogenesis become less strict. This is the case for early ripening varieties characterized by the uncoupling of fruit development and embryogenesis. In fact, at harvest, seed development is still in progress and a long way from maturity. Seed presence is always necessary to achieve normal fruit development even if embryo development is incomplete [5]. Apart from the above observations, molecular-genetic information on the relationship between fruit and seed development is scarce. Cross-talk between the two organs may involve different components of the signaling network, such as hormones, transcription factors (TFs) and other signaling molecules, playing either direct or indirect roles.

Concerning hormones, parthenocarpic fruit development in some species is induced by applications of auxin or cytokinins (CKs), or gibberellins (GAs), or hormone blends [6]. Molecular approaches have confirmed the role played by hormones, especially auxins [7]. Investigations in *Arabidopsis *identified a mutant, named *fwf *(fruit without fertilization), with a normal silique development even in the absence of seeds [8]. Double mutant analysis (*fwf ga1-4, fwf gai, fwf spy, fwf ats*) pointed out that *FWF *negatively affected GA biosynthesis and GA and auxin signal transduction. The FWF protein may interact with TFs such as Fruitful (FUL) and Aberrant Testa Shape (ATS), members of the MADS-box family, and Scarecrow -SCR- type, which are all involved in cell division [8]. Additional TFs have been identified, some of which are related to hormone action, actively transcribed along peach fruit development and ripening ([9,10]). Orthologues of these TFs are also expressed in true (silique and berry) and false (pome and strawberry) fruits, supporting the hypothesis that different fruit types share common regulatory elements [11]. High throughput analysis conducted in *Arabidopsis *showed that some TFs are shared by seed and fruit [12].

Taking this information into account, peach seed and fruit transcriptomes were explored throughout development by means of a massive gene approach based on the use of the μPEACH 1.0 array platform and quantitative real time RT-PCR (qRT-PCR). The research identified genes marking organ/tissue developmental phases, as well as candidate signals (hormones and TFs) that may trigger the cross-talk between fruit and seed.

## Results

### Seed and fruit growth pattern

**Figure 1:**
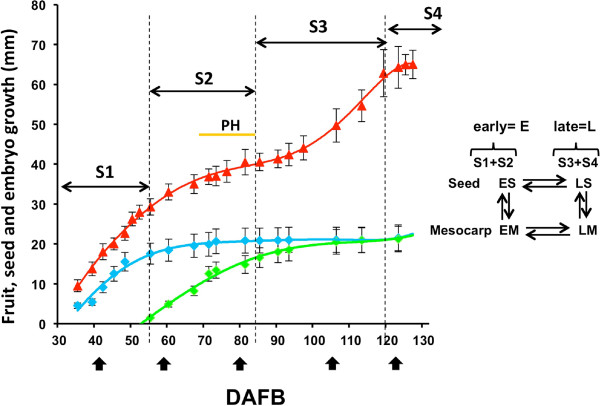
**Fruit and seed growth pattern (cv Fantasia)**. Fruit growth (red) is expressed as cross diameter while length is used for seed (blue) and embryo (green) development. Difference in length between seed and embryo represents endosperm, integuments and nucellus being a minimal part of the seed. Fruit developmental cycle has been divided into 4 main stages (S1 to S4) according to the first derivative of the fruit growth curve. The yellow horizontal line indicates pit hardening. Sampling dates are marked by black arrows. The simple loop microarray experimental design is outlined on the right. For the microarray expression analyses, seed (S) and mesocarp (M) tissues at S1 and S2I, and S3 and S4 were pooled, and defined as early (ES and EM) and late (LS and LM) development, respectively. The comparison has been made between different developmental stages (LS/ES and LM/EM) within the organs and between the two organs (ES/EM and LS/LM) within the developmental stage.

Fruit growth analysis was performed on cv Fantasia and assumed as a reference (Figure 1). In this genotype fruit development and ripening are completed in 135-140 days after full bloom (DAFB). Growth dynamics display the typical double sigmoidal pattern in which four developmental stages have been identified according to the first derivative. S1, S2, S3 and S4 lasted for 45, 32, 33 and 17 days, respectively. Pit hardening (PH) started 60 DAFB and was completed by the S2/S3 transition. The seed derives from the fertilized ovule and the initial increase in length (Figure 1) is due to the rapid nuclear division of the endosperm responsible for embryo sac expansion. Endosperm cellularization starts 40 DAFB and is completed by the beginning of PH. The embryo develops very slowly in the early stages (S1 and S2), reaching a length of about 40-60 μm. Later, at the S2/S3 transition, it resumes development reaching its final size by the middle of S3. The morphological completion of development is followed by maturation and desiccation.

### Identification of marker genes

RNAs extracted before (E, early development) and after (L, late development) pit hardening have been used for microarray transcriptome analyses in order to identify genes possibly involved in seed-pericarp cross-talk or useful as organ and developmental phase molecular markers. Data obtained from the microarray analyses were handled either as single comparisons, i.e. late seed vs. early seed (LS/ES), late mesocarp vs. early mesocarp (LM/EM), within each hybridization or by combining the whole set of data, thus also including ES/EM and LS/LM (see Figure 1 insert). The microarray expression data (see Additional file 1), validated by means of qRT-PCR on 29 randomly selected genes, showed a Pearson correlation coefficient ranging, in the four comparisons, from 0.79 to 0.84 (see Additional file 2).

**Figure 2:**
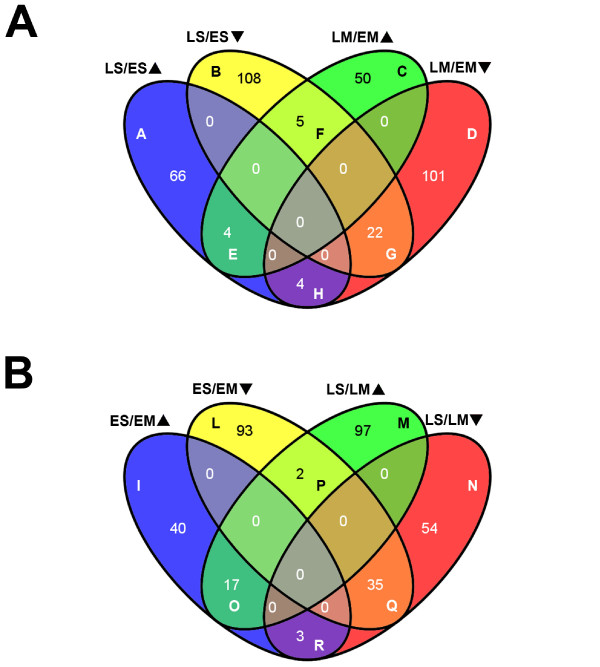
**Genes differentially expressed according to the developmental stage of the organ**. Venn diagrams were used to visualize genes differentially expressed in the microarray experiments. Comparisons between early (E) and late (L) development (panel A), and seed (S) and mesocarp (M) (panel B), were made by means of a direct comparison approach (LS/ES and LM/EM in A; ES/EM and LS/LM in B). Arrowhead orientation indicates up (▲) and down (▼) regulation. The letters inside the sectors are tags for the identification of the genes listed in Additional file 1.

With the single comparison analyses, among the 360 differentially expressed genes within the two organs at early and late development (Figure 2A), 174 and 151 were differentially expressed only in seed (groups A and B) and mesocarp (groups C and D), respectively. Of the seed differentially expressed genes, 108 and 66 were more transcribed at early (group B) and late development (group A), respectively. Four genes, shared by seed and mesocarp, were more actively transcribed at late development (group E), while an additional four showed the opposite trend of expression, being induced in LS and repressed in LM (group H). In addition to the 108 genes more abundantly transcribed in ES (group B), 22 were also expressed in EM (group G), while 5 were abundant in ES and EM (group F). Among the mesocarp differentially expressed genes, 101 and 50 were more transcribed in EM (group D) and LM (group C), respectively. Taking the comparison between seed and mesocarp (ES/EM and LS/LM) into account, 341 genes were differentially expressed in the two organs (Figure 2B). Among these, 133 and 151 were differentially expressed only at early (groups I and L) and late (groups M and N) development, respectively. Considering the differentially-expressed genes at early development, 40 mRNAs were more abundant in seed (group I) and 93 in mesocarp (group L), while among the late development ones, 97 were more abundant in seed (group M) and 54 in mesocarp (group N). Of the 57 remaining genes, 17 and 35 transcripts were always more abundant in seed (group O) and mesocarp (group Q), respectively, and 5 displayed an opposite pattern, being more (3) or less (2) abundant in ES (group R) and ML (group P). Annotations of genes included in Figure 2 are reported with microarray expression data in Additional file 1.

**Figure 3:**
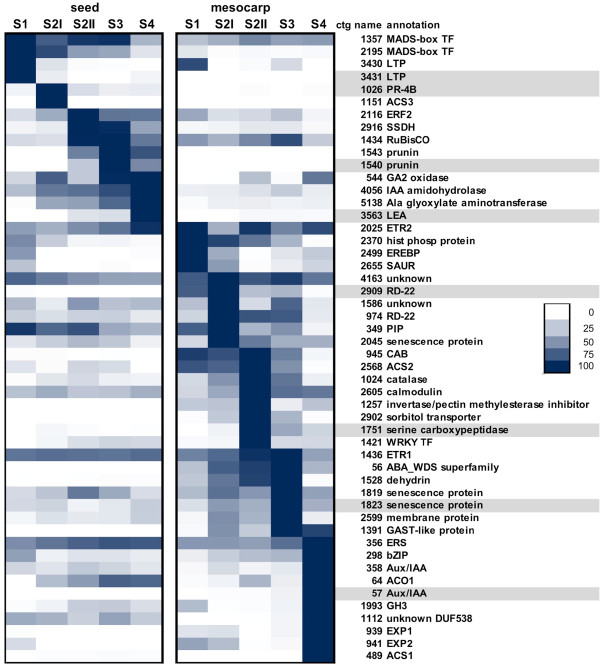
**Selection of developmental stage and organ specific marker genes**. Identification of putative marker genes was performed by selecting some of those differentially expressed in the microarray analyses and further validated by means of qRT-PCR. This detailed expression profiling allowed the selection of those genes that best fitted the ideal marker characteristics as indicated in the Methods section. Expression profiles of 50 genes were measured in seed and mesocarp at five different developmental stages (S1 to S4). Expression values, as indicated in the insert, are related to the highest expression of each gene (100% blue). Genes have been manually ordered according to their expression profiles. Grey shading highlights genes selected as markers.

Based on the above microarray analysis, putative markers were searched to find those that meet the following criteria: a) moderately to highly expressed in only one organ (seed or mesocarp), b) highly expressed/not expressed at specific developmental stage/s (S1 to S4). According to these criteria, 50 potential marker genes, chosen among those differentially expressed in the microarray, were selected and tested by means of qRT-PCR in leaf, flower (data not shown), seed and mesocarp at five developmental stages in cv Fantasia (Figure 3). These detailed expression profiles allowed the identification of eight genes best fulfilling the ideal marker criteria. For seed development, ctg3431, coding for a lipid transfer protein (LTP), ctg1026, coding for a pathogenesis related (PR) protein, ctg1540, coding for a prunin, and ctg3563, coding for a late embryogenesis abundant (LEA) protein, have been chosen as S1, S2, S3 and S4 markers, respectively. Concerning mesocarp development, ctg2909, coding for a RD22-like protein, ctg1751, coding for serine carboxypeptidase, ctg1823, coding for a senescent associated protein, and ctg57, coding for an AUX/IAA protein, have been selected as S1, S2, S3 and S4 markers, respectively (Figure 3). The function as stage markers has been confirmed on the same genotype for an additional growing season (Additional file 3).

**Figure 4:**
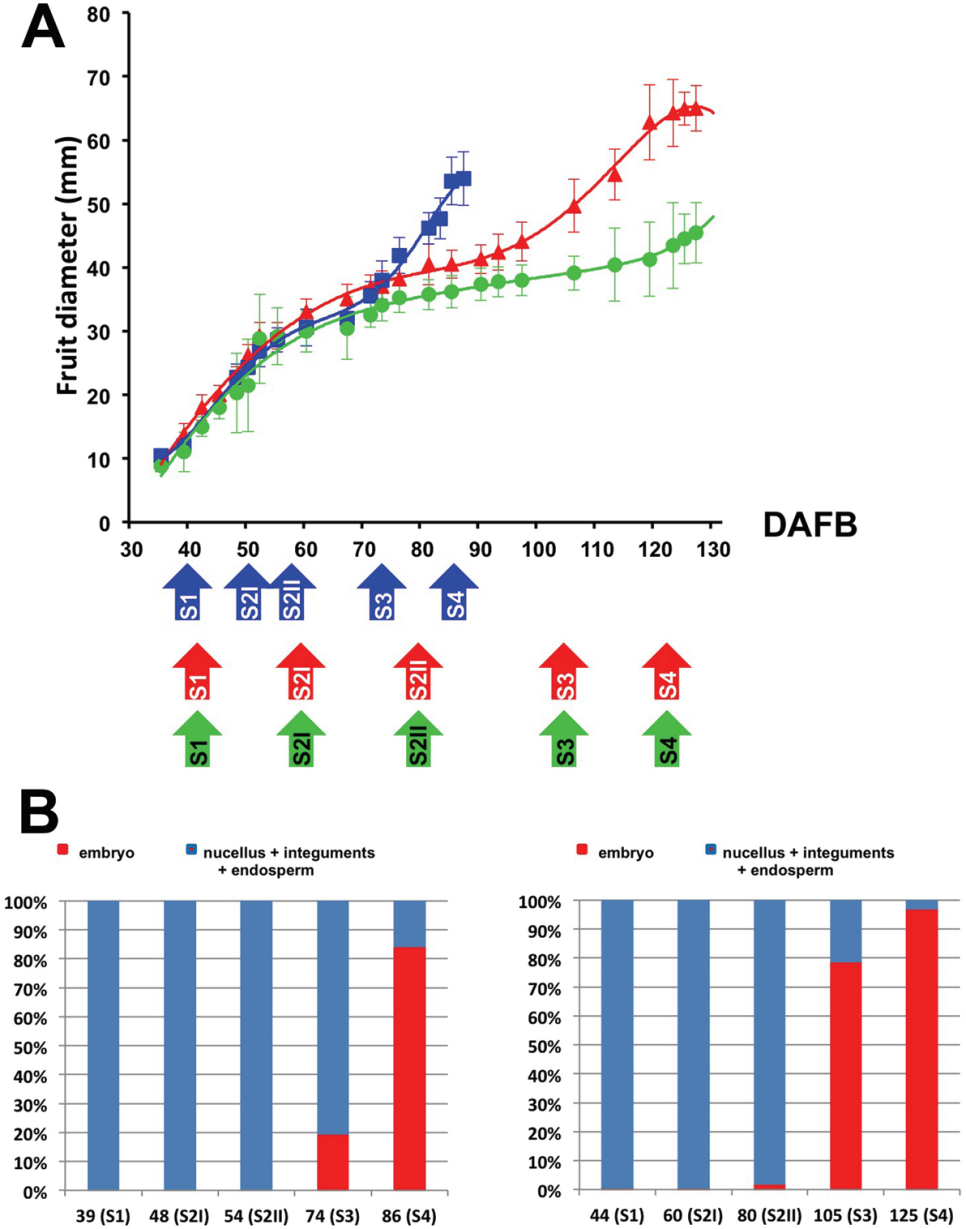
**Dynamics of fruit and seed growth in Fantasia, Springcrest and *slr***. A) Fruit growth curves are expressed as cross diameter (mm) for Fantasia (the reference genotype; red triangles), Springcrest (the early ripening genotype; blue squares) and **slr **(the slow ripening genotype; green circles). In the lower part of the panel, the arrowheads indicate the timing of sampling for the 3 cvs and the developmental stage is indicated within each arrow. B) Dynamics of seed development in Springcrest (left) and Fantasia (right) related to the fruit developmental stages. Seed development in **slr **is similar to that reported for Fantasia. Relative abundance of nucellus, integuments and endosperm (blue) and embryo (red) points out that in Springcrest, at fruit harvest, embryo development is a long way from maturity, while in **slr**, in spite of the block of fruit ripening, the completion of embryo development parallels that of Fantasia and the seed is viable.

A further validation of the selected genes was performed in two additional genotypes (cv Springcrest and the *slow ripening *- *slr *- selection) differing for the dynamics of seed and fruit development. In Springcrest, fruit ripening occurred after 86 DAFB (Figure 4A), when seed development was still in progress (Figure 4B). At the end of the growing season (taking cv Fantasia as a reference), *slr *showed a fully developed seed (Figures 4A and 4B), while the mesocarp development was blocked at stage S3.

**Figure 5:**
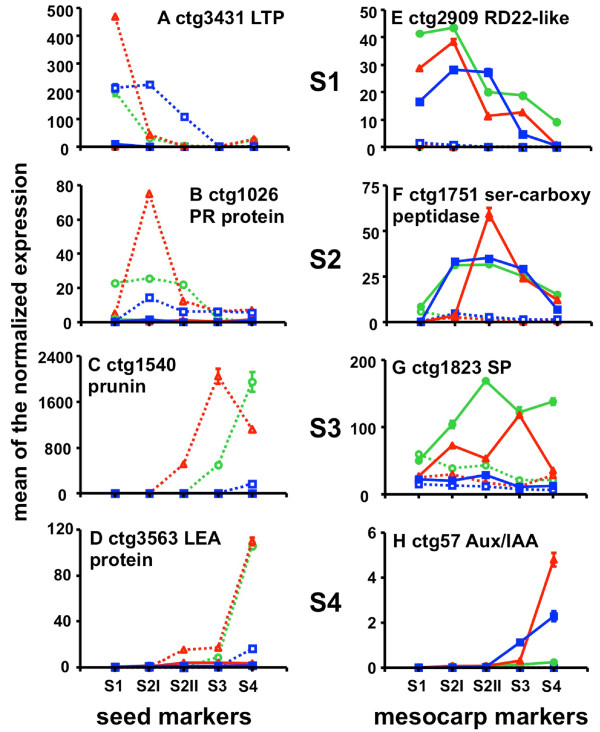
**Validation of developmental stage and organ specific markers in mesocarp and seed of three genotypes**. Expression pattern, assessed by qRT-PCR, of seed (dashed lines) and mesocarp (solid lines) molecular markers of Fantasia (red triangles), Springcrest (blue squares) and **slr **(green circles), at five developmental stages (S1 to S4). Transcript levels are measured as means of normalized expression ± SEM of three technical replicates.

As regards seed markers, ctg3431, coding for a LTP, clearly marked the S1 stage for both Fantasia and *slr*, while in Springcrest its expression decreased only at S3 stage (Figure 5A). A PR protein encoding gene, ctg1026, has been selected for the S2 stage. The highest expression level was found in the seed of cv Fantasia, peaking at early S2 and decreasing thereafter, as in Springcrest. In *slr*, its expression was broader, being relevant also at S1 and S2II (87 and 86% of S2I, set as 100%, respectively; Figure 5B). A prunin, the main seed storage protein in *Prunus *spp., encoded by ctg1540, is a good marker for S3 seed development only in Fantasia. In fact, different amounts and kinetics of its transcript accumulation were observed in the other two genotypes. In Fantasia, accumulation started between S2I and S2II and increased up to a maximum at S3, decreasing thereafter, whereas in *slr *and Springcrest transcript accumulation was delayed, becoming detectable at S3 in the former and S4 in the latter (Figure 5C). The expression of the gene encoding a LEA protein (ctg3563) became detectable at S2II in Fantasia and peaked at S4. A similar pattern was observed in *slr*, although the transcript only started to be detectable at S3. In Springcrest, it was detectable only at S4, at levels lower than in the other two genotypes (Figure 5D). The level of expression of the four genes in mesocarp was very low throughout development and comparable in the three cvs (Figures 5A-D).

As regards mesocarp, ctg2909, coding for an RD22-like protein, had maximum expression at S1 and early S2 (i.e. S2I, Figure 5E). In Fantasia and *slr *the expression decreased already at S2II (28% and 32% of the maximum in Fantasia and *slr*, respectively), while in Springcrest its expression was still high (96%) at S2II.

A serine carboxypeptidase (ctg1751) was chosen as a marker for the S2 developmental stage. In Fantasia, the transcript was undetectable at S1, at basal level at S2I, peaked sharply at S2II, and then declined at S3 and S4. Also in the other two varieties the transcript was undetectable at S1, but its expression, already high at S2I, slightly increased at S2II and remained at high levels at S3, decreasing at S4 (Figure 5F). The expression of ctg1823, encoding a senescence related protein, had a maximum in Fantasia at S3 (100%), while expression levels were much lower in the previous and following stages (29 and 9% at S2II and S4, respectively). Although its expression was relatively high (50%) at S2I, it may be considered a good S3 marker. In Springcrest, the expression was generally low at all stages, with a maximum at S2II. In the *slr *genotype, the accumulation of ctg1823 transcripts steadily increased during the early phases up to a maximum at S2II. Although slightly decreasing thereafter, the ctg1823 mRNA was also abundant at S3 and S4 (60 and 74% of S2II, respectively) (Figure 5G). S4 stage is clearly identified by the expression of ctg57, coding for an Aux/IAA protein. In Fantasia, the expression at S3 is about 6% of that measured at S4 and almost undetectable in early phases. In Springcrest its expression is also almost undetectable at S1, S2I and S2II, but at S3 it is already half of that measured at S4. In *slr*, although maximum expression is at S4, the transcripts accumulated at very low levels (5% of Fantasia) (Figure 5H).

In agreement with their being mesocarp markers, all the selected genes are almost undetectable in seed (Figure 5E-H) with the exception of ctg1823 in *slr*.

### Hormones and TFs in seed fruit cross-talk

Hormone-related genes possibly involved in cross-talk between the two organs were identified among those spotted on the microarray based upon the list of hormonal indexes available for *Arabidopsis *([13]; TAIR website). The portion of hormone responsive genes in *Arabidopsis *ranges between 3.8 and 9.4% of the whole transcriptome (TAIR 10 vers., 27,416 genes), depending on the hormone considered (Additional file 4). For μPEACH 1.0 (4,806 targets), the portion of hormone responsive genes parallels that of *Arabidopsis*, ranging from 3.8 to 9.8% with values for each hormone class comparable to those calculated for *Arabidopsis*. An irrelevant bias may therefore be assumed to exist when peach expression data are used for HORMONOMETER analysis [13]. In addition, it could be assumed that the same proportion might be expected if a whole genome array were used.

**Figure 6:**
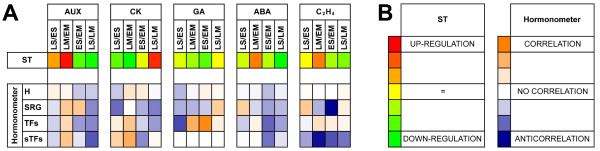
**Heat map showing the relationship between the expression of signal transduction and hormone target genes**. **Panel A**. The heat map was produced by considering the genes involved in the signal transduction (ST) for auxin (AUX), cytokinin (CK), gibberellic acid (GA), abscissic acid (ABA) and ethylene (C*_2_*H*_4_*). HORMONOMETER data were grouped into hormone-responsive genes (H), genes with hormone-specific responsiveness (SRG), hormone-responsive genes encoding TFs (TFs), and genes encoding TFs with hormone-specific responsiveness (sTFs). For each hormone, the following comparisons have been analyzed: LS/SE, LM/EM, ES/EM and LS/LM. **Panel B **Color codes for ST genes and hormone-responsive genes (HORMONOMETER). For ST, red and green represent up- and down-regulation, respectively. In the HORMONOMETER, orange (value = 1), white (value = 0), and blue (value = -1) indicate a complete correlation, no correlation, or anti-correlation, respectively, in terms of direction and intensity of the hormone index with the queried experiment [13].

A heat map was produced by considering the following subsets of genes for each hormone (Figure 6): i) genes involved in signal transduction (ST), ii) hormone-responsive genes (H), iii) genes with hormone-specific responsiveness (SRG), iv) hormone-responsive genes encoding TFs (TFs), and v) genes encoding TFs with hormone-specific responsiveness (sTFs). The subset i) was identified using the classification of *Arabidopsis *orthologs obtained from TAIR GO terms and AHD classification lists (available at http://ahd.cbi.pku.edu.cn/; [14]), and was then analyzed by averaging the log ratios, while the other subsets were used for the HORMONOMETER analyses [13].

Concerning auxin and intra-organ comparisons (LS/ES and LM/EM), a weak activation of ST was observed in LS with respect to ES, paralleled by a partial correlation with the overall reference hormone indexes, whereas a partial anti-correlation was observed when auxin-specific hormone indexes, TF- and specific TF-encoding targets were used. In the mesocarp, a marked up-regulation of ST subset was evidenced in LM, and a good correlation was shown in the same sample both considering the overall hormone indexes and all the other gene subsets. As regards inter-organ comparisons, a decreased transcription of ST elements was always observed in the seed, paralleled by an anti-correlation with the overall hormone indexes at both early (ES/EM) and late (LS/LM) development. However, considering the specific subset, a slight correlation was found in the former comparison, whereas all the results in the latter one were consistent with the overall HORMONOMETER data.

The intra-organ comparison LS/ES indicated a down-regulation of cytokinin (CK) ST elements at late seed development, paralleled by an anti-correlation with both the overall and specific hormone indexes. However, a slight correlation was observed in terms of specific TFs, while all TFs appeared not correlated. Concerning the mesocarp, a lower activation of ST elements in LM than EM was counteracted by a strong correlation with CK indexes. CK-specific genes appeared not correlated, whereas TFs showed a slight correlation, becoming stronger when only the CK-specific TFs were considered. As regards inter-organ comparisons, a low activation of the signal transduction in ES was counteracted by a strong correlation with overall hormone indexes. When the analysis was performed with the other subsets, a significant anti-correlation was observed. Finally, during late seed development, despite the higher activation of ST elements compared to the mesocarp, a general anti-correlation was shown, with the exception of specific TFs, which appeared not correlated.

Considering the gibberellins (GAs)-related expression data, the LS/ES comparison demonstrated a good consistency in signal transduction, and anti-correlation with overall and specific transcriptional indexes, and TFs, except for the GA-specific TFs, that were not correlated. The mesocarp profile was similar except when all TFs were considered, the latter analysis showing a robust correlation. In the ES/EM inter-organ comparison, a depression of the ST pathway in the seed was evidenced. The overall HORMONOMETER analysis showed no correlation with GA hormone indexes, whereas an anti-correlation resulted from the analysis of hormone-specific targets. When all the TFs underwent the HORMONOMETER analysis, a strong correlation was shown, while specific TFs were not correlated. The most significant data pointed out by the LS/LM comparison concerned the analysis of GA-specific indexes, showing a slight correlation.

As regards abscisic acid (ABA) and intra-organ comparisons, in spite of a down-regulation of its ST pathway during late seed development, a correlation was observed in terms of both overall and ABA-specific indexes. TFs were basically anti-correlated and not correlated, when considered either as a whole or just the specific ones, respectively. In the mesocarp, despite a weak up-regulation of the ST elements found in LM, there was no significant correlation in any of the HORMONOMETER analyses. Moving to inter-organ comparison ES/EM, the down-regulation of signal transduction occurring in ES paralleled an anti-correlation found in all the gene sets. In the LS/LM comparison, similar results were obtained in terms of both signal transduction and HORMONOMETER.

Concerning ethylene, no variation was observed between LS and ES in terms of expression of genes encoding ST elements. In spite of this, a slight correlation was pointed out by both overall and ethylene-specific gene targets. Moreover, TFs were not correlated, while specific TFs were slightly anti-correlated. With the LM/EM comparison, the hormone signaling pathway was up-regulated in LM, paralleled by a partial correlation of TFs. On the other hand, both the hormone specific subsets showed an anti-correlation, stronger in the case of TFs. Both inter-organ comparisons (ES/EM and LS/LM) displayed a down-regulation of the ST pathway in the seed. The HORMONOMETER analyses showed no correlation when all targets and all TFs were considered, and anti-correlation concerning the specific targets and TFs, stronger for the former. Both signal transduction and HORMONOMETER results related to jasmonates, salicylic acid, and brassinosteroids are presented and discussed in Additional file 5.

## Discussion

This research was mainly focused on the relationship between seed and pericarp throughout development, using a mass gene approach by means of the μPEACH1.0 [9]. Although this platform was developed mainly from late development mesocarp cDNAs, hybridization analyses and differential expression profiles assessed for both early developing mesocarp and seed indicate that μPEACH1.0 is also a reliable tool for these transcriptomic investigations.

Concerning marker genes, morphological observations pointed out that the dynamics of seed development in different genotypes is quite synchronous, whereas a wide variation exists in the pericarp, affecting not only the length of the developmental phases but also important traits related to fruit quality, such as the degree of endocarp lignification (cartilaginous endocarp), flesh texture (melting/non-melting), sugar/acid ratio, etc. Accordingly, the singling out of marker genes specific for the same developmental stage is not always unequivocal for all three studied genotypes. Moreover, since seed sampling was referred to the fruit developmental stages (S1, S2, S3 and S4), expression data should be read taking into account the uncoupling that exists between seed and fruit development in Springcrest, an early ripening cultivar.

The ctg3431, marking S1 in the seed, encodes a lipid transfer protein similar to *Arabidopsis *LTP1 [15]. Its gene expression profile in peach is consistent with *Arabidopsis *data, the latter showing that *LTP1*, along with *LTP3*, *LTP4 *and *LTP6*, is expressed at high levels during early seed development [16]. The function of this gene as an S1 marker was confirmed in all the genotypes. The delayed decay of transcript accumulation assessed in the seed of cv Springcrest has, in fact, to be related to the acceleration of mesocarp development in this genotype (Figure 4). The ctg1026 (Figure 5B) is similar to a carrot PR which has been related to early embryo development, being expressed in the endosperm and secreted in the apoplast, thus positively regulating embryo fate and patterning [17]. It is interesting to note that in cv Springcrest, the down-regulation of ctg1026 at S3 and S4 occurs at a slower rate than in Fantasia and *slr*, thus confirming the uncoupling of seed and mesocarp development also at the molecular level. The different kinetics observed for the expression of S3 marker, a gene encoding a prunin storage protein (ctg1540, Figure 5C) in *slr *indicates that in this selection, as well as the blocked development of the mesocarp, some variations in seed storage accumulation may also exist. The apparent delay in transcript accumulation measured in Springcrest is again due, as in the case of ctg3431, to the uncoupled development of seed and pericarp. Ctg3563, encoding a LEA (late embryogenesis abundant) protein, is a very reliable marker of S4, in both Fantasia and *slr*, indicating that the seed can reach a fully matured stage in both genotypes. The very low levels of *LEA *gene expression detected at S4 in Springcrest are consistent with the uncoupling that exists between seed and pericarp maturation in this genotype.

Concerning the mesocarp, ctg2909, marking S1 and S2I, encodes a putative RD22-like protein, whose expression in both *Arabidopsis *and grape is partially under the control of ABA and claimed to be involved in stress responses [18,19]. Since the levels of this hormone in peach mesocarp were shown to follow a biphasic pattern with two peaks at S2I and S4 [20], the increasing expression of ctg2909 at early mesocarp development might be related to the level of ABA. However, while the hormone also peaks at S4, the expression levels of this gene did not, thus indicating a dual regulatory mechanism triggering its expression, possibly also under a developmental control as shown in the seed of *Arabidopsis *[19]. The delayed decay of ctg2909 expression observed in Springcrest might be related to the higher growth potential of this early ripening variety documented by the S2 phase length, which is significantly reduced compared to cv Fantasia (Figure 5E). The S2 phase is marked by ctg1751 (Figure 5F), coding for a serine carboxypeptidase (SCP). SCPs are members of the α/β hydrolase family of proteins, claimed to function also as acyltransferases and lyases in the biosynthesis of secondary metabolites [21]. Taking into account that the most important event occurring at S2II is endocarp lignification, an indirect role in this process might be hypothesized for ctg1751. Ctg1823 (Figure 5G) was shown to be a good S3 marker in Fantasia, but not in *slr *and Springcrest. Since this gene encodes a putative senescence-associated protein, a likely failure of the senescence process and/or of its entry phase may be hypothesized in the two genotypes in which mesocarp development is either slowed down or accelerated. Interestingly, when mesocarp development is slowed down, as in *slr*, the peak of expression of ctg1823 is anticipated, whereas in the other case (*i.e*. in Springcrest), in which mesocarp ripens very rapidly, the peak is almost absent. It may be speculated that an overly precocious start of senescence would not allow the fruit to shift from maturation to ripening [22], and, vice versa, an acceleration of fruit ripening is achieved if senescence is not initiated. For the S4 phase, a very reliable marker is represented by ctg57 (Figure 5H), coding for an already partially characterized peach Aux/IAA protein [10]. Its expression was shown to increase at early S4, most likely under a developmental control, thereafter decreasing when ethylene climacteric is fully installed. Accordingly, ethylene treatments were shown to reduce the specific transcripts. Besides fully agreeing with previous data, the expression profiles shown here may also represent correlative evidence for a putative functional role. Indeed, no rise of expression was measured in the mesocarp of *slr*, consistent with the block/slowdown of development and ripening. Moreover, in the case of Springcrest, a high ethylene-producing variety [23], the rise in expression of ctg57 is both anticipated, paralleling ripening kinetics, and less pronounced than in Fantasia, in agreement with a negative effect exerted by higher levels of ethylene.

Possible mechanisms involved in seed-pericarp cross-talk should take into account the vascular and cellular connections existing between the two organs. It has been shown that all the maternal tissues of pericarp and seed (integuments) are intensively interconnected (Vizzotto, personal communication), while nucellar tissue is excluded from the plasmodesmata network. This implies that the flux of metabolites, as well as signaling molecules between embryo and fruit, must occur through the apoplast. Taking into account that hormones play a pivotal role in the regulation of seed and fruit development, it has been assumed that they might also be involved in the cross-talk between the two organs. The heat map data (Figure 6) will therefore be discussed taking into account the consistency of the colors in the following main two-by-two comparisons: ST/H, SRG/H, TFs/H, and sTFs/SRG. More specifically, considering the first one (ST/H), consistency of colors may indicate a relationship between the hormone-related response and activation of the corresponding signal transduction pathway. In the second comparison (SRG/H), the same parameter may provide information about the hormone specificity of the transcriptional response, and, at the same time, of the possible cross-talk between hormones. A double comparison (TFs/H, and sTFs/SRG) may allow it to be pointed out if other players besides the TFs are involved in the regulation of the downstream processes, and if a specific response is mediated by hormone-specific TFs. Auxin, cytokinins, and gibberellins are generally considered to be the most relevant hormones for early seed and fruit development, whereas abscisic acid and ethylene play important roles in seed maturation and fruit ripening. From the point of view of the cross-talk between seed and mesocarp, comparisons should refer to the same developmental stage, i.e. ES/EM and LS/LM. Concerning auxin, the data presented here point out that the specificity of the response to the hormone is higher in ES and LM, although the relationship between the overall HORMONOMETER (H) and ST data indicates that mesocarp is always more sensitive than the seed to the hormone. Taking into account that the presence of a viable seed is required for fruit set and development in peach [2], and that the overexpression of auxin biosynthetic genes in the ovary stimulates the parthenocarpic fruit development in several species [6], it may be hypothesized that the signal produced by the developing seed might be either the auxin itself exported to the fruit, as demonstrated in tomato [24], or a secondary messenger whose target at the fruit level includes a large subset of auxin-responsive genes. This is consistent with both the high specificity of the auxin response shown here in the early developing seed and the higher sensitivity to the hormone displayed by the mesocarp paralleled by a strong hormone response. Among the mesocarp auxin responsive genes, several encode elements regulating transport (ctg2448, ctg2449 and ctg2789 [25] Additional file 1), indicating that auxin movement in this tissue is a relevant process, thus strengthening the hypothesis that auxin produced by the seed may behave as a signal efficiently transported to and within the mesocarp. An Aux/IAA-encoding gene (ctg358) showed an opposite transcription profile in the two organs, being abundant in ES and LM. It has been demonstrated that its tomato orthologue (i.e. LS-IAA9, [26]) acts as a repressor of auxin signaling. Thus, its expression in young organs (low in mesocarp, high in seed) seems to confirm that the hormonal response is not at the synthesis site. Finally, the expression of ctg2655, a SAUR-like IAA responsive gene [27] was found to be higher in mesocarp than in seed (see also Figure 3), thus suggesting a higher auxin level in EM than in ES [28,29].

The main process regulated by CKs is cell division, occurring at early development in both seed (endosperm) and mesocarp. In the former, there is an up-regulation of signal transduction elements, such as ctg2370 coding for a histidine-containing phosphotransfer protein [30] whose transcription is abundant in very young organs (Figure 3). The corresponding substantial activation of hormonal targets, including several CK-specific genes, might differ in the two organs. For example, a cellulose synthase (ctg3673) is activated in EM but not in ES, cytokinesis being an LS event, whereas cyclin D3 (ctg779) was up-regulated in both organs at the early stage. However, this transcriptional response did not just involve CK-specific TFs, implying that other regulatory elements may determine the hormone-specific gene activation. A similar activation of signal transduction elements to that found in the seed is present in the mesocarp at early development. However, the overall and the CK-specific target activation are not correlated to the hormone action, suggesting that CKs may regulate mesocarp cell division at the post-transcriptional level [31], either alone or in cooperation with other phytohormones. Moreover, considering the inter-organ comparison, it is noteworthy that during early development the seed displayed a higher sensitivity to CKs than the mesocarp but a lower specificity of response. The amount of the overall transcriptional response observed in the seed may be due to the involvement of other hormones besides CKs [32]. During late development, an inverse situation was observed compared to the early phases. In fact, the high activation of signal transduction pathways occurring in the seed was uncoupled from the overall transcriptional response, which was even more specific in the mesocarp. The CK-mediated up-regulation of genes encoding sorbitol dehydrogenases (ctg636 and ctg1378, Additional file 1) appears particularly interesting, as this might increase the sink strength of the seed and attract photoassimilates to the entire fruit, which become more competitive in the partitioning process [33].

From a physiological point of view, GAs play a stimulatory role in fruit development, as shown by the ability to induce parthenocarpy in several species [34] when applied in post-bloom phase and/or early development. The initial phases of endosperm and embryo development are usually related to a high level of GAs [35], while seed maturation is paralleled by a decay of free GAs and increase of their conjugates. The HORMONOMETER data confirmed these results both in seed and mesocarp, except for TFs in the latter. In fact, the most relevant transcriptional response occurred during early development at seed level as pointed out by the ES-specific expression of ctg3431 (Figure 5) encoding an orthologue of the *Arabidopsis *LTP1 (AT2G34580), which is classified as a GA-responsive gene (see at http://genome.weizmann.ac.il/hormonometer/) involved in embryo patterning [36]. In the mesocarp, a low correlation was observed between the TF-related transcriptional response and GA action, implying the activation of complex regulatory mechanisms that may play relevant roles in the cross-talk between seed and mesocarp. A possible mode of interaction might be the EM specific expression of a gene coding for a Zinc finger protein (ctg187), whose *Arabidopsis *orthologue (AT2G04240, XERICO) interacts with DELLA proteins, is repressed by GA, and causes ABA accumulation when over-expressed [37]. However, since this transcriptional response lacked specificity, it might be hypothesized that GA action also depends on the interaction with other hormones. It has recently been demonstrated that auxin induced parthenocarpy via GAs in unpollinated tomato ovaries [38]. Furthermore, the peculiar expression profile of ctg1391, encoding a GAST-like protein, orthologue of *Arabidopsis *GASA6 (AT1G74670), in EM is confined to S2 and S4 stages, when cell enlargement is slow (Figure 3). These data are in agreement with the observed inhibition of cell elongation conferred on both *Arabidopsis *seedling and strawberry fruit over-expressing the *Fragaria *orthologue *FaGAST *[39]. During late development, in spite of the slight correlation existing in terms of GA-specific response, the other gene sets appeared not to be correlated to the hormone action. It may be deduced from this that the role of GA in the cross-talk between seed and mesocarp is negligible during late development.

ABA is known to play an antagonistic role with respect to auxin, GAs and CKs, as observed during fruit development in avocado [40] and tomato [41,42]. According to the HORMONOMETER, this antagonism was largely confirmed in the seed, the transcriptional response being correlated with higher levels of the hormone in LS compared to ES, also when the ABA-specific subset was considered. In fact, during late seed development, ABA levels are known to increase and GA-related genes such as ctg3430, encoding a LTP-like, are down-regulated (Figure 3 and Additional file 1). This physiological parameter is paralleled by a consistent transcriptional response in which TFs belonging to WRKY (ctg1545), HD (ctg499), Aux/IAA (ctg768), bZIP (ctg 724) and DREB-like AP2 (ctg 4674) families are involved. Given this interpretation and taking into account that during both early and late development ABA ST pathways and ABA-target responses are more active in the mesocarp, the hormone may play a more relevant role in the development of each organ, rather than in seed-mesocarp cross-talk. In this context, the ABA pool of maternal and zygotic origin may trigger independent transduction pathways.

The well-known role of ethylene in peach ripening [9,10] was confirmed by the higher level of transcription of its ST elements (ctg4109, ctg244 and 4757 coding for an ETR2-like ethylene receptor and two ERFs, respectively) measured at the mesocarp level during late development. It is worth noting that ethylene-related transcriptional response in the LM/EM comparison resembles that of ABA, most likely because of the significant number of transcriptional targets shared by the two hormones (50 out of 216 ABA- and 235 ethylene- responsive genes). This was not observed in the inter-organ assessments, in which, differently from ABA, a weaker overall transcriptional response was pointed out, with the only exception being ethylene-specific TFs that were more represented in mesocarp. As regards cross-talk, the role of ethylene might be limited to the very early phase of fruit development, as demonstrated in tomato [43]. This might be a consequence of the fact that ethylene acts mainly within the cell where it is synthesized. Also in this case, the hormone of maternal and zygotic origin may activate independent pathways controlling different processes.

## Conclusions

In this research, genes were identified marking different developmental phases of seed and mesocarp. The reliability of these molecular markers was tested in two subsequent years and a further functional validation was carried out in three different genotypes. In the latter case, data indicate that, while seed markers represent reliable tools in all the tested varieties, in the case of the mesocarp the different developmental and ripening traits of the various genotypes somewhat affect the expression of marker genes, consistently with their putative functions and cv characteristics. The most critical phases, from the point of view of mesocarp marker retrieval, were S2II and S3. This might be related to the high divergence in pericarp development among the different genotypes, as pointed out above. However, this limitation may be partially overcome by using mesocarp markers as a whole, therefore increasing their discriminating power.

As regards the cross-talk between seed and pericarp, possible candidate signals were identified among hormones. In the early phases, when the cross-talk is more vital for fruit set, the candidates are auxin, CKs, and GAs, acting either directly (auxin) or indirectly as signals, whereas ABA and ethylene appear to be involved later on.

Further investigations relying upon the availability of whole genome platforms will allow enrichment of the marker genes repertoire and elucidation of the cross-talk mechanisms between the two organs, taking into account, besides hormones, other players such as hormone peptides and microRNAs.

## Methods

### Plant materials

Fruit growth analysis was conducted on peach trees of cv Fantasia grown on the experimental farm of the University of Padova (Legnaro), Italy, as described by Tonutti et al., 1997. Fruits from 10 trees were collected at 42, 60, 81, 106 and 123 days after full bloom (DAFB), corresponding to the first exponential growth phase (S1), the onset (S2I) and end (S2II) of pit hardening, second exponential growth phase (S3) and ripening (S4), respectively. For each sampling, mesocarp and seed were excised from 30 fruit, pooled in three biological replicates and then immediately frozen in liquid nitrogen and stored at -80°C until use. To monitor seed development, seeds were excised from fruit at weekly intervals from late S1 to ripening. Seed and embryo length were measured by stereomicroscopy [44].

Seed and fruit of two additional genotypes (cv Springcrest and selection *slow ripening *- *slr*-) characterized by uncoupling of seed and fruit development, were used for the validation of marker gene functions. In Springcrest, an early ripening cultivar, fruit ripening occurs when seed development is still in progress. In fact, seeds become viable only after *in vitro *cultivation. *slr *is a selection obtained from a free-pollinated population of Fantasia, characterized by a block of mesocarp development at stage S3 but with a fully developed seed. Sampling of mesocarp and seed was performed throughout fruit development as previously described.

Fruit growth analyses were performed in 2008 and repeated in 2010; the array experiments were performed on samples collected in the former year, whereas expression data were validated by qRT-PCR on samples of both years. Only data related to 2008 are presented and discussed in the paper. Data from 2010 are given in the supplementary material (Additional file 3).

### Transcriptome analysis

For each sampling data, total RNAs were extracted, as described in [45], from each of the three biological replicates of seed and mesocarp and stored at -80°C for transcriptional analysis.

To elucidate the interactions between seed and mesocarp, a mass gene approach was followed by using the μPEACH1.0 as described in [10]. Comparisons were made by pooling stage 1 and 2 (named early development, E), and stage 3 and 4 (named late development, L), separately for mesocarp (M) and seed (S), and using a simple loop experimental design (Figure 1).

Data were analyzed using the TM4 software platform [46] as previously described [10]. A SAM (Significance Analysis of Microarrays [47]) analysis was performed to identify significantly differentially expressed genes using a False Discovery rate of 0% (90^th^%ile). Among these, up and down regulated genes were identified assuming a threshold ratio of expression as log_2 _higher than 1 and lower than -1, respectively.

To improve the annotation of targets spotted on the μPEACH1.0 platform, all the oligo sequences were blasted against a transcript dataset obtained by assembling 280,000 454 reads (Additional file 7) with the about 90,000 sanger *Prunus persica *ESTs present in the NCBI database. The 454 reads have been obtained from a normalized library constructed by pooling equal amounts of mesocarp RNA from stages S1, S2, S3 and S4 (GenXPro GmbH, Germany). The 32,162 new contigs present in this new database have been compared to those used to develop the μPEACH1.0 platform and, if longer than the old ones, used for BLAST analysis. Contigs (Additional file 8) were analyzed by BLAST against already classified proteins from *Arabidopsis *(TAIR 10 release) to categorize them by using the GO terms developed by TAIR http://www.arabidopsis.org/portals/genAnnotation/functional_annotation/ontologies.jsp for the biological processes ontology. Based on the best BLAST search results and using a cut-off e value of 1*e-^10^, the peach genes were assigned to the categories according to the most similar *Arabidopsis *genes (Additional file 1).

Differentially expressed genes were visualized with Venn diagrams drawn with Venny [48], clustered according to their expression profiles by using the Quality Threshold Clustering (QTC) coexpression algorithm [49] and grouped in four main charts allowing intra and inter-organ as well as developmental-stage comparisons.

The data discussed in this paper have been deposited in NCBI's Gene Expression Omnibus and are accessible through GEO Series accession number GSE22582 http://www.ncbi.nlm.nih.gov/geo/query/acc.cgi?acc=GSE22582.

qRT-PCR was performed and the obtained data manipulated as previously described [10]. Briefly, 3 μg of total RNA for each sample, pre-treated with 1.5 units of DNaseI, was converted to cDNAs by means of the "High Capacity cDNA Archive Kit" (Applied Biosystems), which uses random examers as primers. Primer sequences for the selected genes are listed in Additional file 6. Oligonucleotides PpN1for (CCAGGAGAATCGGTGAGCAGAAAA) and PpN1rev (TCGAGGGTGGAGGACTTGAGAATG) annealing to the peach putative transcript ppa009483 m, orthologous to *Arabidopsis *AT4G34270, were used to amplify the reference gene. The peach reference gene was selected starting from *Arabidopsis *homologous genes [50], tested for transcript normalization in peach (Tadiello and Trainotti, unpublished results) and chosen to normalize qRT-PCR data because of its superior result compared to the previously used Internal Transcribed Spacer of the ribosomal RNA [10]. Reactions were performed using 10 μL of the "Syber green PCR master mix" (Applied Biosystems), with 0.05 pmoles of each primer, in the "7500" instrument (Applied Biosystems). The obtained CT values were analyzed by means of the "Q-gene" software ([51]), averaging three independently calculated normalized expression values for each sample. Expression values are given as mean of the normalized expression values of the triplicates, calculated according to equation 2 of the "Q-gene" software ([51]). Differences in expression values among probes reflect different quantities of target amounts. Numerical values obtained with these calculations were transformed into graphics or used to build heat maps with MS Excel.

### The hormonometer analysis

The HORMONOMETER http://genome.weizmann.ac.il/hormonometer/ is a bioinformatic tool for assessing any transcriptome response according to the perspective of similar events occurring upon hormonal activation [13]. A vector-based correlation is calculated by comparing the variation of the transcriptome in a query experiment with an indexed list of pre-calculated transcriptional responses established by published hormone treatments in *Arabidopsis *[52]. Input data, for each gene, consist of the fold change calculated by directly dividing the normalized expression values measured for the two samples to be compared, and the respective P-value of its significance. When the variations detected in the query resemble those of the reference pool related to a certain hormone, it is assumed that the same hormone may have caused the transcriptional response observed in the query. In the HORMONOMETER output data, the numeral 1 indicates a complete correlation in terms of direction and intensity of the hormone index with the queried experiment, 0 indicates no correlation, and -1 indicates the highest possible anti-correlation for each transcript in the index [13]. Given that input data for each gene derive from a two-by-two comparison (for example, sample A versus sample B), correlation and anti-correlation indicate higher levels of the active hormone that are transduced into a measurable transcriptional response in either sample A or B, respectively, whereas no correlation (the numeral 0) indicates that the levels of the hormone are the same in both samples.

Since HORMONOMETER only accepts *Arabidopsis *data as input along with their corresponding locus name and Affymetrix probe IDs, the putative *Arabidopsis *orthologues of peach genes, obtained by using the best BLAST hit of the updated μPEACH1.0 database against TAIR 10, were used as input data with peach expression values. In addition to the whole set of peach genes, three subsets were submitted to HORMONOMETER: i) genes with hormone-specific responsiveness (i.e. that are not multiple targets of hormones), ii) hormone-responsive genes encoding TFs, and iii) genes encoding TFs with hormone-specific responsiveness.

## Authors' contributions

CB, LT, ARa devised the study and participated in its design and coordination; FZ conducted the microarray experiments; ARi collected fruit material, measured seed development parameters and performed the validation of microarray data by qRT-PCR; AT performed the validation of microarray data by qRT-PCR; CB, LT, AB, VZ and ARa analyzed the data; CB, AB, LT, ARa and GC wrote the paper. All authors read and approved the final manuscript.

## Supplementary Material

Additional file 1 **Average microarray signal values for all the μPEACH1.0 probes**. Column headings are as follows: A: contig_name; B: Operon ID (probe identification of the manufacturer); C: oligo sequence; D: Annotation source (sequences used to identify the best TAIR 10 BLAST HIT. "Original contig" means that the sequence used comes from the older database, while "454" indicates that a new sequence, longer than the original one, has been used); E: source ID (database from which sequences used for the annotation. Nucleotide sequences are available as additional file 7 ("454") and 8 ("Original contigs"); F: TAIR 10 best hit (against μPEACH1.0 transcripts); G: Sequence description (annotation of the TAIR 10 best hit); H: μPEACH1.0 original annotation; I to P: mean and standard deviation of the mean of normalized signal values in the different comparisons (ES/EM, LS/LM, LS/ES, LM/EM); Q and R: tags for the identification of genes included in the Venn diagrams (Figure 2); S: probes positive to SAM in all 4 comparisons. From column T to AA: genes involved in hormone metabolism and signaling for auxin, CKs, GAs, ABA, ethylene, jasmonate, salicylate and brassinosteroids are marked. Click here for file

Additional file 2 **Correlation between microarray and qRT-PCR expression values**. The qRT-PCR expression values of 29 genes, listed in Additional file 6, were plotted against the microarray hybridization signals and correlation indexes (Pearson coefficient is reported in the inserted rectangles) have been calculated separately for each direct comparison (LM/EM, blue diamonds; ES/EM, purple squares; LS/ES, green triangles; LS/LM, light blue circles) (panel A). The validation for 21 randomly selected contigs is shown in the panel B. Click here for file

Additional file 3 **Validation of seed and mesocarp markers in the 2010 season**. Expression profiles of seed (panel A) and mesocarp (panel B) selected markers throughout fruit development. The expression values are given as a percentage distribution throughout development of the total gene expression (100%). Fisher's Least Significant Difference (LSD) was calculated for each gene time series using "agricolae" R package (de Mendiburu Felipe, A statistical analysis tool for agricultural research. MS thesis. Universidad Nacional de Ingenieria, Lima-Peru. 2009). Panel A: ctg3431 LSD = 8.6%, ctg1026 LSD = 11.2%, ctg1540 LSD = 15.1%, ctg 3563 LSD = 7.6%. Panel B: ctg2909 LSD = 4.8%, ctg1751 LSD = 4.6%, ctg1823 LSD = 5.4%, ctg57 LSD = 3.8% Click here for file

Additional file 4 **Suitability of μPEACH1.0 for the HORMONOMETER platform**. Table 1: Number and percentage of putative hormone-related genes spotted on the μPEACH1.0 microarray. Table 2: Number of genes representing putative common targets of different pairs of hormones as assessed in *Arabidopsis *[13]. The total number of hormone-related genes is given in bold on the diagonal. Click here for file

Additional file 5 **Heat map showing the relationship between the expression of signal transduction and hormone target genes**. **Panel A**. The heat map was produced by considering the genes involved in the signal transduction (ST) for jasmonic acid (JA), salicylic acid (SA), and brassinosteroids (BR). HORMONOMETER data were grouped into hormone-responsive genes (H), genes with hormone-specific responsiveness (SRG), hormone-responsive genes encoding TFs (TFs), and genes encoding TFs with hormone-specific responsiveness (sTFs). For each hormone, the following comparisons have been analyzed: LS/SE, LM/EM, ES/EM and LS/LM. **Panel B **Color codes for ST genes and hormone-responsive genes (HORMONOMETER). For ST, red and green represent up- and down-regulation, respectively. In the HORMONOMETER, orange (value = 1), white (value = 0), and blue (value = -1) indicate a complete correlation, no correlation, or anti-correlation, respectively, in terms of direction and intensity of the hormone index with the queried experiment [13]. Click here for file

Additional file 6 **List of the oligonucleotides used for the qRT-PCR analyses**. The "Contig_nos" refers to the peach contig numbers in the database used to prepare the oligo probes of the μPEACH1.0 microarray. Click here for file

Additional file 7 **Sequences of 454 reads**. Nucleotide sequences of 454 reads. Click here for file

Additional file 8 **Sequences of Contigs**. Nucleotide sequences of contigs. Click here for file
