# Supplementary figures and images for: A microarray approach to identify genes involved in seed-pericarp cross-talk and development in peach
Source: BMC Plant Biol. 2011 Jun 16;11:107. doi: 10.1186/1471-2229-11-107 (PMC3141638; doi:10.1186/1471-2229-11-107)

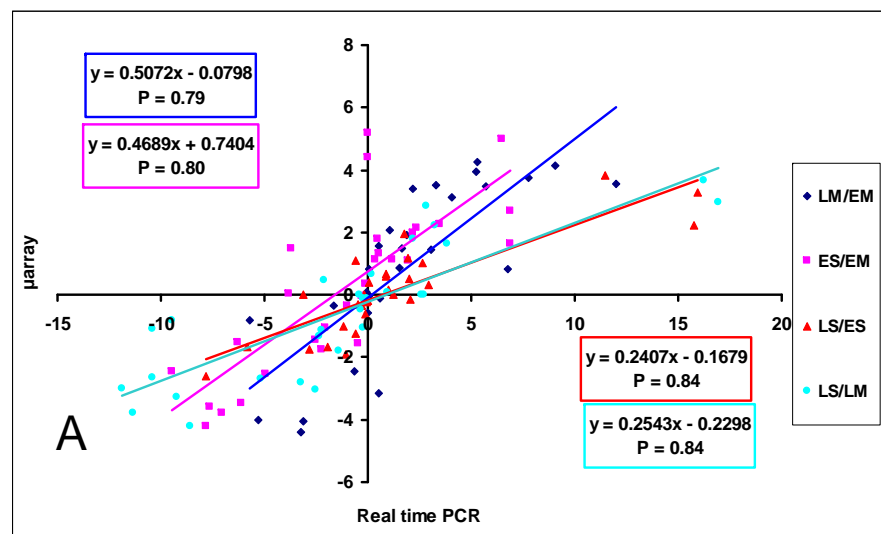

**B**

quantitative real time PCR

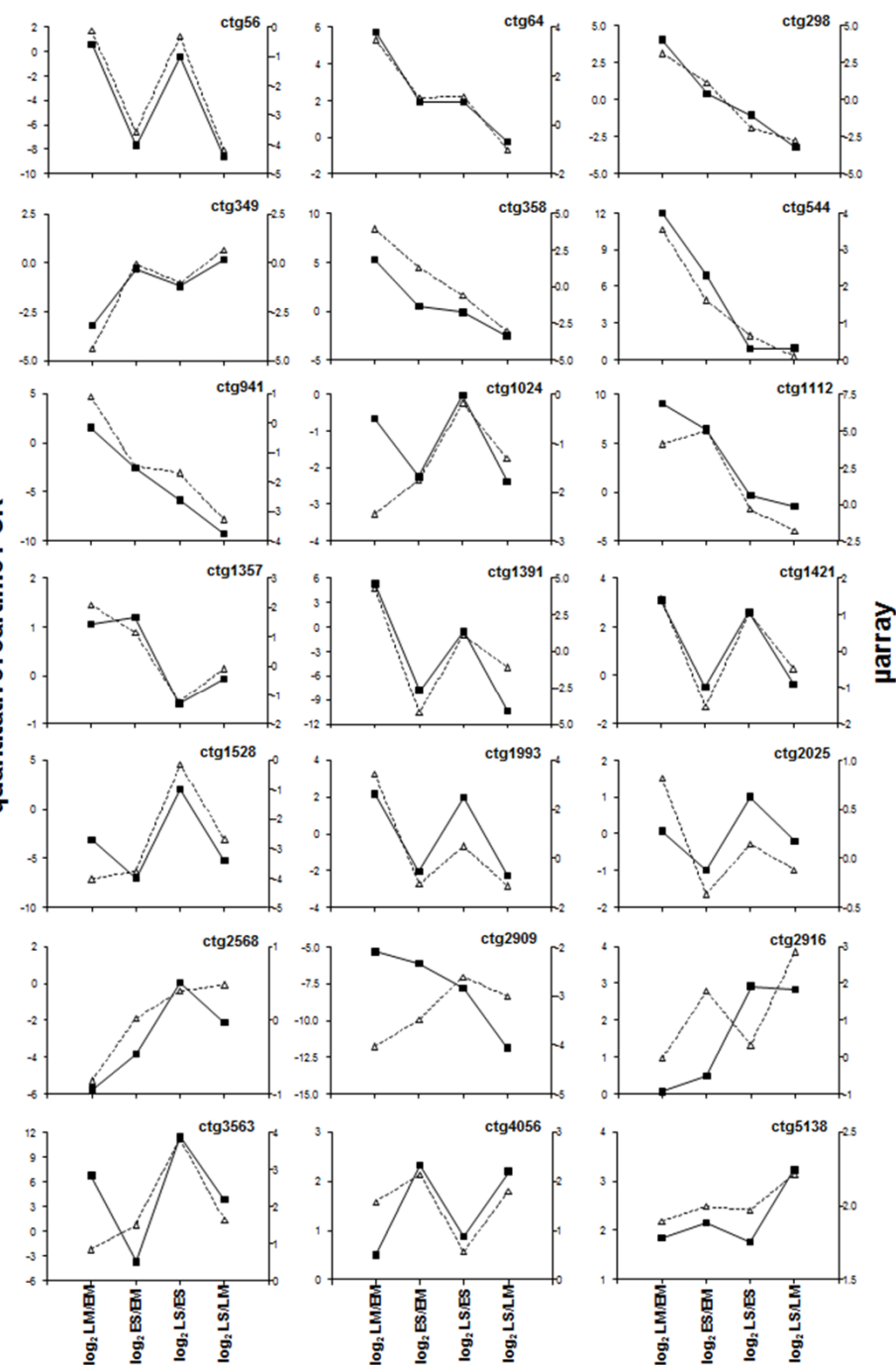

Supplement: Additional file 2 — Correlation between microarray and qRT-PCR expression values. The qRT-PCR expression values of 29 genes, listed in Additional file 6, were plotted against the microarray hybridization signals and correlation indexes (Pearson coefficient is reported in the inserted rectangles) have been calculated separately for each direct comparison (LM/EM, blue diamonds; ES/EM, purple squares; LS/ES, green triangles; LS/LM, light blue circles) (panel A). The validation for 21 randomly selected contigs is shown in the panel B. [file 1471-2229-11-107-S2.PDF]

A

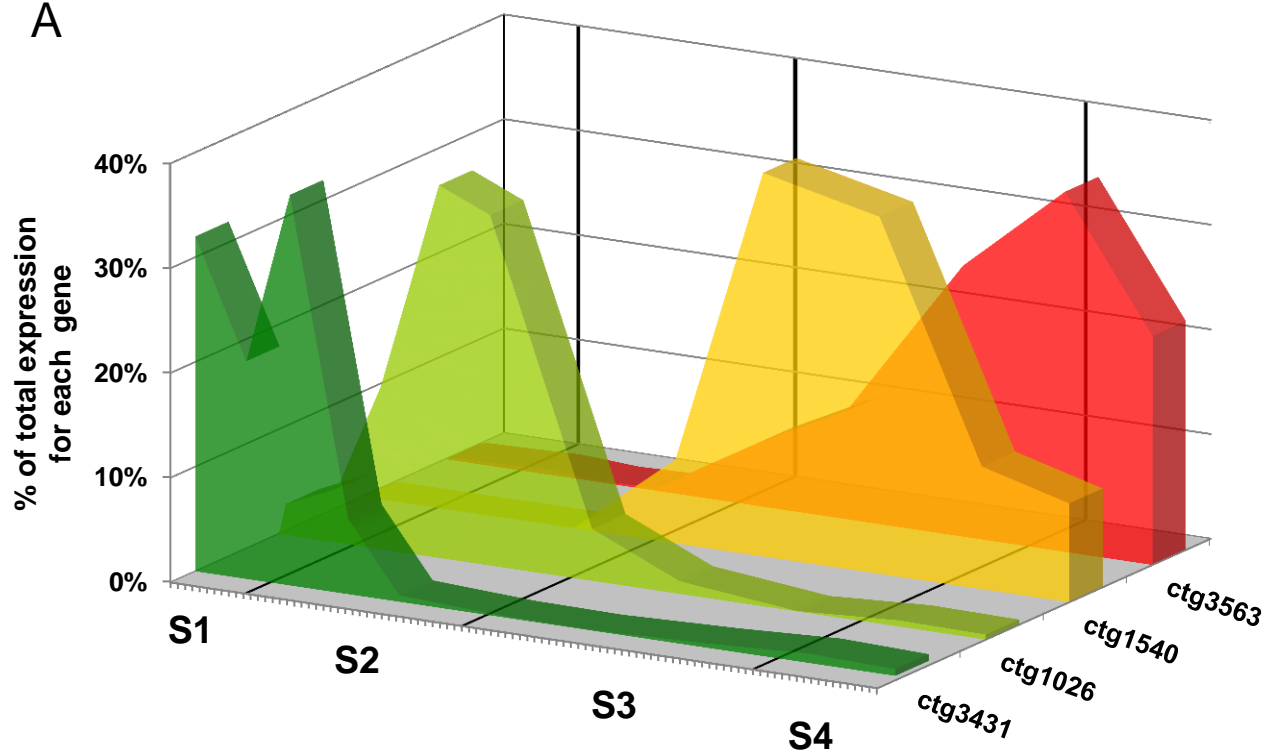

B

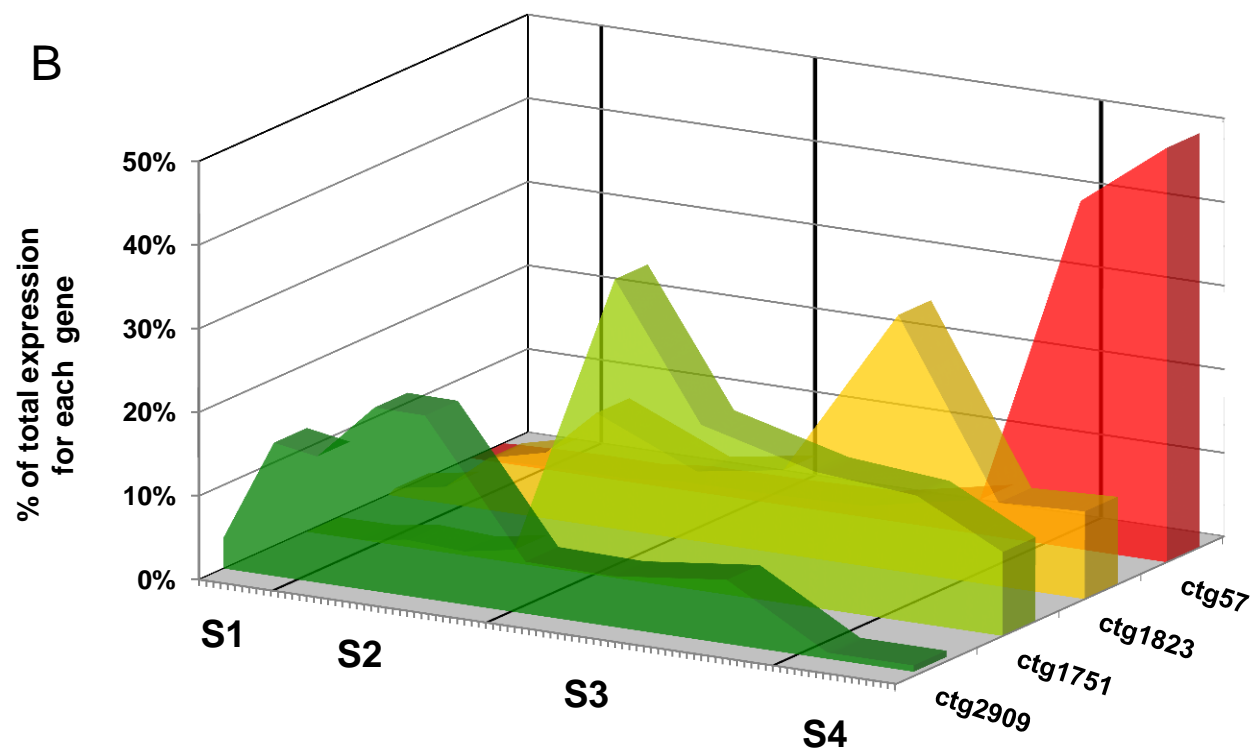

Supplement: Additional file 3 — Validation of seed and mesocarp markers in the 2010 season. Expression profiles of seed (panel A) and mesocarp (panel B) selected markers throughout fruit development. The expression values are given as a percentage distribution throughout development of the total gene expression (100%). Fisher's Least Significant Difference (LSD) was calculated for each gene time series using "agricolae" R package (de Mendiburu Felipe, A statistical analysis tool for agricultural research. MS thesis. Universidad Nacional de Ingenieria, Lima-Peru. 2009). Panel A: ctg3431 LSD = 8.6%, ctg1026 LSD = 11.2%, ctg1540 LSD = 15.1%, ctg 3563 LSD = 7.6%. Panel B: ctg2909 LSD = 4.8%, ctg1751 LSD = 4.6%, ctg1823 LSD = 5.4%, ctg57 LSD = 3.8% [file 1471-2229-11-107-S3.PDF]
