# Supplementary material for: A microarray approach to identify genes involved in seed-pericarp cross-talk and development in peach
Source: BMC Plant Biol. 2011 Jun 16;11:107. doi: 10.1186/1471-2229-11-107 (PMC3141638; doi:10.1186/1471-2229-11-107)
Supplement: Additional file 4 — Suitability of μPEACH1.0 for the HORMONOMETER platform. Table 1: Number and percentage of putative hormone-related genes spotted on the μPEACH1.0 microarray. Table 2: Number of genes representing putative common targets of different pairs of hormones as assessed in Arabidopsis [13]. The total number of hormone-related genes is given in bold on the diagonal. [file 1471-2229-11-107-S4.PDF]

Table 1 – Number and percentage of putative hormone-related genes spotted on the µPEACH 1.0 microarray.

	**No.** Arabidopsis **hormone indexes (% of the whole transcriptome)**	**Total (% of the whole array)**	**Signal Transduction**	**Response**
Auxin	2,261 (8.3%)	359 (7.5%)	38	364
Cytokinin	2,494 (9.1%)	430 (8.9%)	11	438
Gibberellin	2,330 (8.5%)	381 (7.9%)	13	385
Abscisic acid	2,216 (8,1%)	388 (8.1%)	49	409
Ethylene	2,567 (9.4%)	411 (8.6%)	28	441
Jasmonate	1,901 (6.9%)	313 (6.5%)	20	312
Salicylate	1,037 (3.8%)	184 (3.8%)	21	189
Brassinosteroid	2,568 (9.4%)	472 (9.8%)	17	467

Table 2 – Number of genes representing putative common targets of different pairs of hormones as assessed in Arabidopsis (Volodarsky et al., 2009). The total number of hormone-related genes is given in bold on the diagonal.

	**Auxin**	**Cytokinin**	**Gibberellin**	**Abscisic acid**	**Ethylene**	**Jasmonate**	**Salicylate**	**Brassinosteroid**
**Auxin**	**359**							
**Cytokinin**	103	**430**						
**Gibberellin**	65	88	**381**					
**Abscisic acid**	93	99	60	**388**				
**Ethylene**	109	93	72	101	**411**			
**Jasmonate**	85	102	61	107	99	**313**		
**Salicylate**	26	33	28	35	35	29	**184**	
**Brassinosteroid**	83	91	81	102	135	99	39	**472**
