# Supplementary material for: A microarray approach to identify genes involved in seed-pericarp cross-talk and development in peach
Source: BMC Plant Biol. 2011 Jun 16;11:107. doi: 10.1186/1471-2229-11-107 (PMC3141638; doi:10.1186/1471-2229-11-107)
Supplement: Additional file 5 — Heat map showing the relationship between the expression of signal transduction and hormone target genes. Panel A. The heat map was produced by considering the genes involved in the signal transduction (ST) for jasmonic acid (JA), salicylic acid (SA), and brassinosteroids (BR). HORMONOMETER data were grouped into hormone-responsive genes (H), genes with hormone-specific responsiveness (SRG), hormone-responsive genes encoding TFs (TFs), and genes encoding TFs with hormone-specific responsiveness (sTFs). For each hormone, the following comparisons have been analyzed: LS/SE, LM/EM, ES/EM and LS/LM. Panel B Color codes for ST genes and hormone-responsive genes (HORMONOMETER). For ST, red and green represent up- and down-regulation, respectively. In the HORMONOMETER, orange (value = 1), white (value = 0), and blue (value = -1) indicate a complete correlation, no correlation, or anti-correlation, respectively, in terms of direction and intensity of the hormone index with the queried experiment [13]. [file 1471-2229-11-107-S5.PDF]

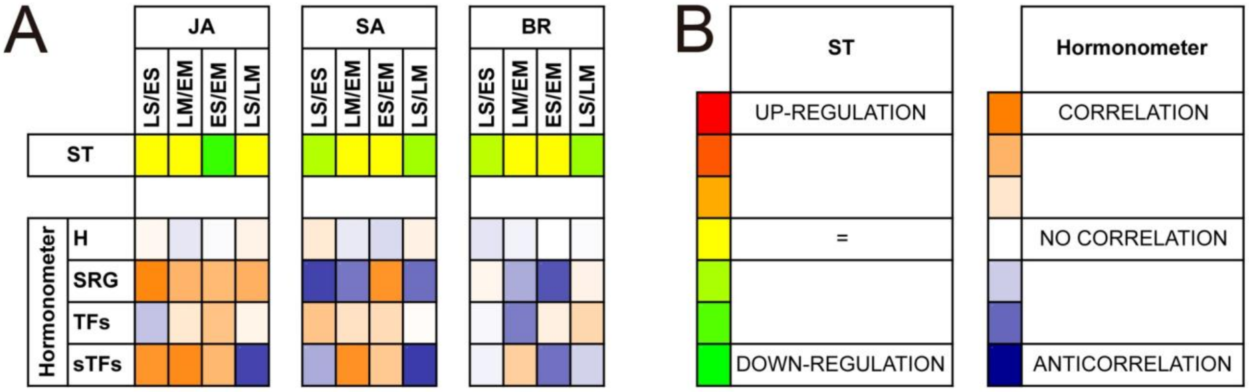

As far as jasmonic acid is concerned, signal transduction (ST) elements were differentially expressed only in the comparison ES/EM, with a down-regulation assessed in the early developing seed. The overall HORMONOMETER analysis pointed out no correlation, whereas considering the hormone-specific target genes (SRG) correlations were shown for all the four comparisons LS/ES, LM/EM, ES/EM and LS/LM, more significant for the first one. This would imply a more active jasmonate-specific response during late development, always higher in the seed than the mesocarp. TFs were anticorrelated in LS/ES comparison, correlated in the ES/EM one, and not correlated in the other two cases. When specific TFs (sTFs) were considered, data were in agreement with those shown for SRG, except for LS/LM for which a marked anticorrelation was assessed.

Considering salicylic acid (SA) ST elements, a down-regulation was shown in LS/ES and LS/LM. Also in this case the overall HORMONOMETER analysis did not show any significant correlation/anticorrelation, whereas for the SRG a clear anticorrelation was pointed out for LS/ES, LM/EM, and LS/LM. For the ES/EM comparison a marked correlation was pointed out. SRG data, taken as a whole, would indicate decreasing SA-specific target activation from ES to EM, LM, and LS. TFs were correlated in LS/ES comparison, whereas no correlation was shown for the others. sTFs were anticorrelated in the LS/ES and LS/LM comparisons, more markedly in the latter one, and correlated in the case of LM/EM and, at a lesser extent, ES/EM.

Concerning brassinosteroids (BR), the ST elements transcription patterns paralleled those assessed for SA. The overall HORMONOMETER showed again no correlation, whereas the other subsets pointed out some differences. SRG showed BR-anticorrelated transcription patterns in LM/EM and ES/EM, and no correlation in the other two comparisons. TFs were anticorrelated in LM/EM and slightly correlated in LS/LM. An inverse situation was reported for sTFs, which were slightly correlated in the former and slightly anticorrelated in the latter. Finally an anticorrelation was assessed also in the ES/EM comparison.
